# Lens on Tropical Sericulture Development in Indonesia: Recent Status and Future Directions for Industry and Social Forestry

**DOI:** 10.3390/insects13100913

**Published:** 2022-10-08

**Authors:** Lincah Andadari, Dhany Yuniati, Bambang Supriyanto, Murniati, Sri Suharti, Asmanah Widarti, Eden Steven, Andi Sadapotto, Bondan Winarno, Minarningsih, Retno Agustarini, Nurhaedah Muin, Wahyudi Isnan, Yetti Heryati, Yelin Adalina, Irma Yeny, Rosita Dewi, Ari Nurlia, Septiantina Dyah Riendriasari, Kun Estri Maharani, Luthfan Meilana Nugraha, Budi Hadi Narendra

**Affiliations:** 1 Research Center for Applied Zoology, Research Organization for Life Sciences and Environment, National Research and Innovation Agency (BRIN), Bogor 16911, Indonesia; 2 Research Center for Behavioral and Circular Economics, Research Organization for Governance, Economy and Community Welfare, National Research and Innovation Agency (BRIN), Jakarta 10340, Indonesia; 3 Directorate General of Social Forestry and Environmetal Partnership, The Ministry of Environment and Forestry (KLHK), Jakarta 10270, Indonesia; 4 Research Center for Ecology and Ethnobiology, Research Organization for Life Sciences and Environment, National Research and Innovation Agency (BRIN), Bogor 16911, Indonesia; 5 Research Center for Society and Culture, Research Organization for Social, Sciences and Humaniora, National Research and Innovation Agency (BRIN), Jakarta 10340, Indonesia; 6 Emmerich Research Center, Jakarta 14450, Indonesia; 7 Faculty of Forestry, Hasanuddin University, Makasar 90245, Indonesia; 8 Center for Standardization of Sustainable Forest Management Instruments, The Ministry of Environment and Forestry (KLHK), Bogor 16118, Indonesia; 9 Research Center for Biomass and Bioproducts, Research Organization for Life Sciences and Environment, National Research and Innovation Agency (BRIN), Bogor 16911, Indonesia

**Keywords:** *Bombyx mori* L., sericulture, non-timber forest product, livelihood, Indonesia

## Abstract

**Simple Summary:**

Sericulture is a labor-intensive agro-industry business that can increase the community’s welfare and support environmental improvement. In Indonesia, silk, as the final product of sericulture, is a potential non-timber forest product (NTFP) that provides benefits to livelihoods and the forest ecosystem. Silk is a fiber produced by the domestic silkworm or mulberry silk moth, *Bombyx mori* L., belonging to the Lepidopteran order, Bombycidae family, probably providing more than 99% of the world’s silk. However, there are many challenges to its development at both upstream and downstream levels, including the availability of quality eggs, optimal and efficient cultivation, pest and disease control, a lack of policy support, unsustainable production, low product quality, and competition with imported products. This paper discusses the recent status and future directions of sericulture development in Indonesia. Improvements in technical and social-economic aspects can support the development of sericulture in Indonesia through increasing productivity in the upstream sector along with conducive downstream policies and governance.

**Abstract:**

The domestic silkworm or mulberry silk moth, *B. mori* L., provides more than 99% of the world’s silk. Silk, as a sericulture product, was first introduced in Indonesia through a trade mechanism and began to develop in 1953. Several factors (economic, ecological, market, and cultural) support sericulture and make it become one of the non-timber forest product priorities. However, the competitive advantages alone have not encouraged the development of prospective sericulture industry in Indonesia yet. This paper is a review of tropical sericulture development in Indonesia. The literature on the development of sericulture in Indonesia between 1989 and 2022 is used to describe conditions related to mulberry cultivation (moriculture), and silkworm rearing (sericulture), as well as the state of socio-economic development, culture, and institutions. Moriculture and sericulture techniques, socio-economic aspects, institutional arrangements, and community motivations are intertwined, creating a challenging atmosphere for sericulture development. There are potential resources, such as exploring quality mulberry production and quality silkworm production through research and development, valuable cultural aspects, and potential stakeholders to build network engagement. Commitment, cooperation, and action from all stakeholders are needed to enhance the development of sericulture in Indonesia. In this context, the central government can play an important role in facilitating multi-stakeholder partnerships in the development of integrated sericulture in Indonesia.

## 1. Introduction

The COVID-19 pandemic has caused the global agenda—e.g., the Sustainable Development Goals (SDGs) for 2030—to come under pressure. This crisis has plunged millions of people into unemployment and dire health conditions resulting in poverty [1,2,3]. In developing countries, including Indonesia, the impact of the pandemic has been more severe, especially in areas where community dependency on forest resources is high due to the limited availability of both on-farm and off-farm jobs [4,5,6]. The situation has been exacerbated by reduced access to inputs, labor, and agricultural land, resulting in a decrease in production, household income, and nutrition [7].

Many attempts have been made by the government to prevent the most severe negative impacts and to boost the local economy by developing several alternative forest-based agribusinesses [4,8]. In Indonesia, an economic recovery program, including social forestry, has been implemented, with the double objective of enhancing consumption and economic productivity [9]. Forests, as natural resource systems, have the potential to provide multiple benefits. In addition to wood products, forests can provide non-timber forest outputs and environmental benefits. NTPs are biological products other than wood of high value, generally obtained from wild biodiversity in natural or human-modified environments [10,11]. Many studies have revealed that non-timber forest products, including ecotourism, small-scale timber enterprises, and environmental services, could play a significant role in social and economic recovery during and after a pandemic [8,12,13]. Non-timber forest products contribute 80% of the social forestry business model in Indonesia [14].

One of the non-timber forest products with the potential to resolve economic challenges during and after a crisis is the natural silk produced by sericulture agribusiness. Sericulture is a prospective and potential activity that can regularly generate higher income [15,16]. Apart from its ability to provide gainful employment and economic improvement for people in rural areas due to the high selling price of its products [15,16], sericulture also plays an important role in preventing the migration of rural people to urban areas in search of employment [17,18]. The silk industry is labor-intensive [19] and so provides employment for 7.9 million people in India and 20,000 weaver families in Thailand [20]. In the context of Indonesia, there are 1200 silk farmers [21] and 4900 weavers [22] involved in silk agro-industry activities. Sericulture agribusiness has been determined to be one of the five NTFP priorities, having the potential to contribute to the economy of the country and tackle poverty [23,24,25].

Sericulture was first introduced to Indonesia through trade in the 10th century; since 1953 [26], it has developed rapidly as it is suitable to the agroclimatic conditions as well as the local culture. Silk yarn production reached its peak in 1971 with a production of 140 tons but then tended to decrease due to several factors [27], such as the low quality of silkworm seeds, pebrine disease attacks, and mulberry plants as feed have begun to be shifted to other horticultural commodities, low prices for silk products, and a lack of support from silkworm production programs and organization.

The silk agro-industry chain is a sequence of activities starting with mulberry cultivation (moriculture) and silkworm rearing. These stages are in the upstream segment and are commonly known as sericulture. The next phase is reeling raw silk from the cocoons and producing the yarn and its derivative products. These products then deliver to the weaving industry for further processing and marketing. Yarn processing, referred to as the manufacturing sector, is the downstream sector of the agro silk industry [21,24].

Several factors support sericulture and make it become one of the NTFP priorities. Economically, the silk industry is an important way of fulfilling domestic as well as export needs, either in the form of cocoons, yarns, or finished goods [19,28]. It is a potential commodity that can significantly contribute to foreign exchange earnings, raise living standards, and reduce poverty, hunger, gender discrimination, and disease risk. Sericulture is mostly developed in rural and suburban areas and hence provides job opportunities for rural communities [29]. In addition, the sericulture and silk industry in Indonesia represents an economically viable rural enterprise for inclusive development, which can effectively have fall-outs in various industrial sectors [20].

Ecologically, the silk industry involves an environmentally friendly production process. The mulberry tree ensures green land covering, soil conservation, and erosion protection and allows for the use of land that is not suitable for other crop cultivation. The silkworm rearing does not cause pollution, CO_2_ emissions are very low, and waste can be easily degraded [30], so it can coexist well with already inhabited areas [31,32]. Silk produced from sericulture is a non-synthetic, renewable, and biodegradable fiber [15]. Furthermore, sericulture also contributes to the improvement of the microclimate of the area of development, which will eventually improve the surrounding environmental conditions [25]. In addition, mulberry cultivation can only be developed in tropical countries, including Indonesia, which has agroclimatic conditions that are suitable for mulberry and silkworm cultivation [33]. Because it has moderate dry and rainy seasons, this country allows mulberry plants to serve as the main food for silkworms to grow and be cultivated throughout the year [34,35,36].

In terms of market prospects, there is still a gap between the annual world demand for natural silk (92,743 tons per year) and the supply of yarn (83,393 tons) [24,25]. Although it is only ranked twelfth among silk yarn-producing countries in the world, Indonesia has a comparative advantage as it produces better quality yarn than other counties [37] and so has the potential to fill the gap. However, filling the market gap is not easy since there are high-quality standards for the international market. At a domestic level, the demand for silk has not yet been met, and hence must be supplied from imported products. The annual need for silk yarn is 500–800 tons, while the national production in 2015 was only about 12.13 tons, 8.95 tons of which were from South Sulawesi [21]. In the current pandemic situation, the need for cocoons as a raw material for domestically produced silk, which was previously imported from other silk-producing countries, such as China, cannot be fulfilled. The above-mentioned characteristics of the silk industry indicate that sericulture has several social, economic, and ecological advantages [25].

However, until now, the competitive advantage of sericulture has not encouraged the establishment of a strong and independent national sericulture industry in Indonesia. During the period 2011–2015, Indonesia continued to experience a decline in the production of both cocoons and silk yarn, correlated with a decline in the natural silk fabric industry in various regions in Indonesia. Some factors causing the decline of the silk agro-industry in Indonesia include: the limited availability of quality silkworm eggs and feed (mulberry leaves), limited skilled labor, the uncertainty of the product selling price [19,38,39], and pest and disease attacks [21]. Apart from those challenges, Sadapotto [27] stated that ineffective policy formulation for sericulture is actually the main factor behind the failure of sericulture in Indonesia due to neglecting institutional factors and focusing more on physical and technical factors in the analysis of problems regarding sericulture. Sericulture industry development involves lots of stakeholders from upstream to downstream. At the upstream level, the development of silk is the responsibility of the Ministry of Environment and Forestry [40]. At the downstream level, activities are under the authority of several relevant stakeholders, including the Ministry of Industry and Trade and the Ministry of Small–Medium Enterprise and Cooperatives. Strong coordination and collaboration among all stakeholders involved in natural silk production are essential for the development of the sericulture industry in Indonesia [21,27].

At the farm level, although it is not a capital-intensive business, sericulture requires sufficient capital to start by buying silkworm eggs, preparing the land to plant mulberries as silkworm feed, and setting up rearing rooms; not all farmers can afford the costs related to these investments. These challenges have led natural silk agribusiness activities to be increasingly abandoned by farmers who tend to choose other farm activities [41].

Various efforts have been made to overcome this situation by developing good-quality silkworm eggs and mulberry seeds and providing technical assistance to silk farmers; unfortunately, these have not provided significant results yet. The key to the successful development of natural silk lies mainly in the support from government policies, the availability of good quality silkworm eggs and feed, the availability of land resources and easy access to adequate funding, as well as training of workers who have skills and expertise in silk cultivation [42].

Many studies have been conducted regarding the development of the silk industry in Indonesia, but most are still partial. An analysis of the gap between existing practices and the strategies that should be developed is needed to determine how to optimize sericulture agribusiness through conducive upstream to downstream policies and governance suited to Indonesian conditions. This paper aims to describe in detail the various development potentials and challenges that should be faced in order to optimally develop sericulture agribusiness.

## 2. Examining Sericulture Development in Indonesia

Indonesia’s sericulture production centers are in several provinces, including West Java, Central Java, and South Sulawesi. South Sulawesi contributes 70–80% of national silk yarn production [21,43,44]. Silk production has been developed in Indonesia since 1953 [26], and mulberry trees began to be cultivated in South Sulawesi in the early 1960s [19].

**Figure 1:**
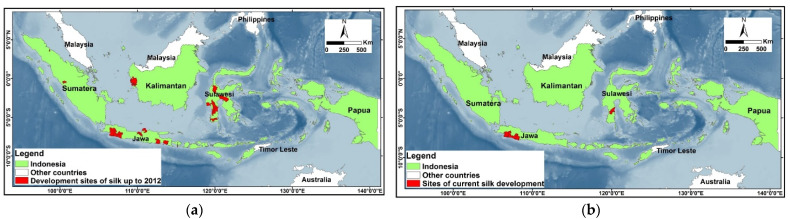
Distribution of Indonesian sericulture development areas. (**a**) before 2012; (**b**) after 2012.

The sericulture in Indonesia has decreased significantly due to the pebrine disease epidemic [21]. Before 2012, the development areas for sericulture were located on the islands of Java, Sulawesi, Kalimantan, and Sumatra, regions that cover 11 provinces. After 2012, the sericultural activity continued in two provinces only, South Sulawesi and West Java (Figure 1).

Various internal and external factors have influenced the decline in the distribution of sericulture development areas; therefore, it is crucial to study the dynamics of sericulture in Indonesia. This section will review the dynamics of sericulture in Indonesia, including the cultivation of mulberry plants (moriculture), rearing of silkworms, products, and social, economic, cultural, and institutional factors.

### 2.1. Cultivation of Mulberry Plant (Moriculture)

Mulberry plants, especially the leaves, are the sole food of the silkworm (*B. Mori* L.) [45,46]. Recently, more attention has been paid to mulberry plant production improvement (quality or quantity)—more than 60% of the total cost of cocoon production goes to mulberry cultivation [45].

Mulberry cultivation in Indonesia uses *Morus cathayana* Hemsl. [47,48,49,50], *M.s alba* L. [47,48,49,50]*, M. nigra* L*., M. multicaulis* (Perr.) Loudon, *M.* *australis* Poir*,* and *M. bombycis* Koidz [47,48,49]. The mulberry species most widely cultivated to date is *M. cathayana* [50]. This species is preferred because it has comparative advantages such as being relatively easy to grow, adaptive to any location, and producing a higher number of leaves compared with other species of *Morus* developed in Indonesia [50]. The scientific name reference of the mulberry species or variety refers to https://powo.science.kew.org/.

In Indonesia, productivity is relatively low, ranging from 8 to 23 tons/ha/year [51,52] compared with in India (the majority of which are *M. alba* var. *multicaulis Loud* and *M. alba* var. *arthoputpurea* (Shahtut)), which reaches 10–30 tons/ha/year in tropical conditions and 25–30 tons/ha/year in temperate regions [53]. China, a major global silk producer, has mulberry productivity ranging from 15 to 46.5 tons/ha/year, depending on the species of mulberry and the conditions of the area of development [54], while production in India ranges from 18.40–23.06 tons/ha/year [55,56].

Mulberry leaves determine the productivity and quality of silkworm products, so we must consider their nutritional quality. The main factors affecting the quality and quantity of mulberry leaves are the genetic factors of each mulberry species, the environment, the cultivation methods, and pest and disease control [57].

Techniques to increase the productivity and quality of mulberry leaves via genetic factors are through type/variety selection and hybridization [47,58,59]. Several studies conducted from 1999 to 2015 in Indonesia have created several mulberry hybrids with 40–53 tons of leaves/ha/year that are resistant to drought, pests, and diseases and are easy to cultivate [60,61]. The crosses came from several sources, including *M. cathayana* Hemsl.*, M. australis* Poir*, M. indica* L*., M. nigra* L*., M. amakusaguwa (Hybrid M. bombycis* Koidz *x M. acidosa* Griff*.), M. acidosa* Griff. and *M. latifolia* Poir. [62,63]. Information on the productivity of mulberry hybridization in Indonesia can be seen in Table 1.

The result of hybridization between *M. cathayana* Hemsl. x *M. amakusaguwa (Hybrid M. bombycis* Koidz *x M. acidosa* Griff*.)*, having the highest leaf productivity, was launched by the Ministry of Forestry in 2013 and named SULI 01 [16]. The SULI 01 mulberry hybrid is suitable to be planted in lowlands and highlands. This accession can produce better quality cocoons than other mulberry varieties and has greater resistance to pests and diseases [64]. Unfortunately, this type of SULI 01 is not well known by the public, so its use as a silkworm feed is still limited. So far, silk farmers have not used hybrid types that have higher productivity than conventional ones.

The environment that affects the productivity of mulberry plants must also be considered. Some of the requirements for mulberry growth are soil conditions that are not loamy, not too sandy, and not acidic; optimal and balanced fertilization; and maintenance according to Standard Operational Procedures (SOPs) at each growth stage and water availability [65]. In Indonesia, the mulberry is found from the lowlands to the highlands (over 3600 m above sea level—m asl). It can grow well even if the leaf production is lower than that of mulberry plants cultivated in areas with an optimum altitude of 400–700 m asl and an optimum temperature of 23.9–26.6 °C [61]. Problems arise because, in extreme areas, silkworm cultivation experiences pose many challenges.

**Figure 2:**
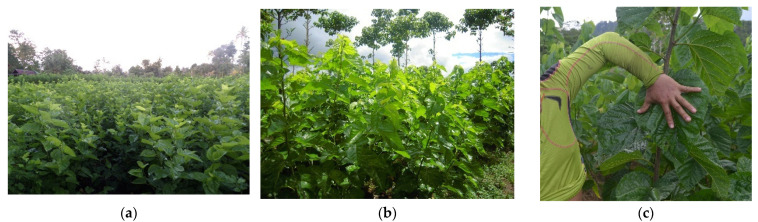
Cultivation of mulberry plants in Indonesia. (**a**) South Sulawesi; (**b**) West Java; (**c**) Hybrid SULI 01.

Although many areas of Indonesia are suitable for the growing of mulberry plants, the tropical climate in Indonesia causes the productivity and supply of mulberry leaves to be discontinuous [28,66]. This is related to the differences in the dry and rainy seasons. Mulberry plants require a lot of water in their growth process, so that in the rainy season, mulberry plants generally grow faster and produce more leaves. Apart from this, the decrease in leaf production in the dry season also depends on the genetic characteristics of the cultivated mulberry varieties [63]. Most of the mulberry plants in Indonesia are propagated vegetatively using stem cuttings, although they can be reproduced by gamic propagation (through seeds). Some mulberry plants in Indonesia can be seen in Figure 2.

Another challenge related to mulberry cultivation is pest and disease attacks. Mulberry plants have some diseases and pests that affect the quality and productivity of the leaves and interfere with the growth and nutrient content in the leaves [67]. Eight types of pests and four types of diseases are commonly found in Indonesia, especially in the central part of South Sulawesi [68], such as grasshoppers (*Valanga nigricornis* (Burmeister), leaf webber (*Glyphodes pulverulentalis* Hampson), snails (*Achatina fulica*), mealybug (*Maconellicoccus hirsutus* Green), white peach scale (*Pseudaulacapsis pentagona* Targioni-Tozzetti), stem borers (*Epepeotes plorator* Newman), powdery mildew (*Phyllactinia corylea* (Pers.) P.Karst), red rust fungus (*Aecidium mori* (Barclay) Barclay), and leaf spot (*Cercospora moricola* Cooke and *Sirosporium mori* (Sydow et P. Sydow) M.B. Ellis). It was stated that these pests and diseases affect the rate of leaf loss up to 15.32% and are influenced by factors related to where the mulberry plants grow. Whitefly (*Bemisia tabaci*) is the most common pest on mulberry plants in Indonesia and can cause damage of up to 80% or crop failure [69]. In India, whitefly attacks cause a leaf yield loss of 4500 kg/ha/year [70].

The use of insecticides and fungicides needs to be controlled because mulberry leaves are used as feed for silkworms in addition to other uses for humans. Due to the application of pesticides over the years, sericulture faces various problems, such as silkworm sensitivity and death. The use of pesticides can increase the mortality of silkworms in the 5th instar. *B. mori* L. silkworms are sensitive to the smell of pesticides, even though they are used for other plants close to mulberry fields or where silkworms are kept [40,71,72]. Pesticides containing dimethoate in mulberry leaf at rearing facilities silkworms will affect cocoon quality, the weight of instar fifth silkworms, and are contained as chemical residues; for this reason, harvest losses are recorded [73].

The area of the mulberry plantation will greatly affect cocoon production. This can be seen in the trend of cocoon production in South Sulawesi Province. Currently, the area of mulberry production in South Sulawesi is 65.2 ha, with cocoon production in two districts (Soppeng and Wajo) where are produced 14,075 kg [40]; in 2014, in the same two districts, there was an area of 238.35 ha of mulberries with cocoon production of 29,249 kg [74]. However, on average, cocoon productivity per hectare of mulberry plants is currently better than in 2014. This is possible because the use of superior eggs of silkworms and mulberry is more intensive and because SOPs for silkworm rearing with supervision extension workers in the area. Other challenges need to be anticipated. This relates to the trend of farmers converting their mulberry plantations into other commodity crops [40,41]. However, they still grow mulberries on part of their land and continue silkworm rearing.

### 2.2. Silkworm Rearing (Sericulture)

Nowadays, the domestic silkworm or mulberry silk moth, *B. mori* L., belonging to the Bombycidae family, provides more than 99% of the silk in the world. Therefore, we can divide silkworm accessions into Chinese, Japanese, European, and Tropical races [75]. However, there are only three accessions of silkworms in Indonesia, Chinese, Japanese and Tropical races.

**Figure 3:**
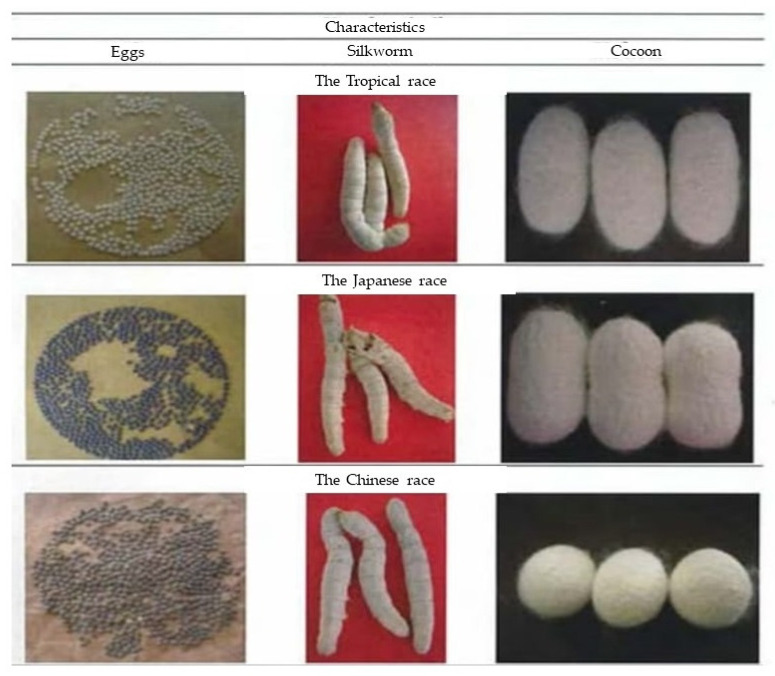
The characteristics of each group of races.

The Chinese race has greenish eggs, yellow eggshells, plain silkworm patterns, blue belly color between segments, shorter silkworm life, oval/oval cocoon larvae shape, and high silk percentage. The Japanese race has gray eggs, white eggshells, heavier eggs, skin spots, pink color between segments of the abdomen, longer silkworm life, stronger silkworm, and peanut-shaped cocoons. Tropical races have white eggshell color, plain silkworm pattern, strong/disease resistant silkworm, short silkworm life, small cocoons, difficult cocoon shells to spin, poor productivity and fiber quality, but high adaptability in Indonesia. The differences in eggs, silkworms, and cocoons of each race in Indonesia can be seen in Figure 3.

In Indonesia, what has been developed are the Chinese race silkworms, the Japanese race, and the result of crossing between the Japanese strains with the Chinese strains [26]. The quality of hybrid silkworm cocoons was better than that of the Chinese and Japanese silkworms [76].

Problems in the rearing of silkworms in Indonesia include: (1) the low quality of silkworm eggs [19]; (2) limited number and supply of silkworm eggs [77]; (3) incompatibility of the type of silkworm with the location of cultivation [78]; (4) inadequate conditions of facilities and infrastructure [78]; (5) lack of farmers’ compliance with good silkworm-rearing standards (SOPs) [79]; and (6) silkworm pests and disease attacks [21]. The limited supply and low quality of silkworm eggs have caused farmers to use the same silkworm eggs for various geographic conditions [78], resulting in varying productivity [77]. Infrastructure and compliance with silkworm rearing standards (SOPs) are important points in silkworm rearing. Silkworm diseases are the most significant factor that impacts cocoon production [21,80,81].

#### 2.2.1. Silkworm Breeding

In general, the silkworm eggs used are of three accessions, coming from three subtropical regions (China, Japan, and Europe), and are usually kept under optimum conditions. The silkworm eggs currently circulating are the result of crossing parents from subtropical areas (bivoltine), apparently not able to adapt well to tropical conditions such as Indonesia [82,83]. For tropical conditions such as those in Indonesia, where the agroclimate fluctuates, the mulberry leaf quality is low, and the silkworm rearing ability is limited, stronger silkworms are needed.

The success of silkworm rearing is highly dependent on the genetic quality of the eggs and their suitability for the environment. Therefore, it is recommended to obtain and use eggs suitable to the conditions of the rearing environment and with the desired properties as targets [84]. Hybridization in Indonesia is mostly carried out by the Sericulture Laboratory under the Ministry of Environment and Forestry and Perum Perhutani (a state forest enterprise). There are five silkworm hybridization products that are developed and used in Indonesia: Hybrid C-301 [52,75,77,85], BS 08 [86], BS 09 [86], PS 01 [87] and SINAR [88]. Information about silkworm hybridization products can be found in Supplementary Table S1.

#### 2.2.2. Silkworm Nursery

After obtaining high-quality silkworm eggs, nursery development is needed so they can be commercialized. Until 2012, Indonesia had only two producers of silkworm eggs, namely Pusat Produksi Ulat Sutera/PPUS (Center for Silkworm Production) Candiroto and Kesatuan Pengusahaan Sutera Alam/KPSA (the Natural Silk Concession Unit) Soppeng [35,86,89,90]. Both institutions are under the management of Perum Perhutani Unit I Central Java (PPUS Candiroto) and Perum Pehutani Unit II East Java (KSPA Soppeng). The production of these two nurseries results from the silkworm hybridization that has been carried out, namely C301 [52,75,77,85] and BS 09 [86]. The following conveys the productivity of silkworm eggs in Indonesia and the institutions that produce them (Table 2).

Hybrid C301 is a silkworm strain widely used in various environmental conditions [39]. Silkworm eggs from two different nurseries reared in the same place sometimes resulted in different productivity [35,86]. Rahmathulla [92] confirmed that seasonal differences in the environmental components significantly affect the genotypic expression in the form of the phenotypic output of silkworm crops, such as cocoon weight, shell weight, and cocoon shell ratio.

The average rearing carried out by each silk farmer is one box containing 25,000 eggs. The average cocoon production in Indonesia from the rearing of one box is 25.03 kg, while the cocoon production in China, the highest producer in the world, can reach 39.97 kg/box [19]. The productivity of the C301 egg hybrid is 25–35 kg/box [74,84], that of the BS 09 hybrid is 27–30 kg/box [74], and that of the PS 01 hybrid is 35–40 kg/box [74,84]. The productivity of the PS 01 hybrid silkworm was better than that of the BS-09 hybrid, which is reared at an altitude of 250 m above sea level; as can be observed according to the body features, mortality of the instar first–four instar silkworms, and the quality of the silkworm cocoons [93].

The number and quality of eggs have experienced ups and downs, thus encouraging importing [94]. In general, silkworm eggs are imported from China. This can be seen from the source of silkworm eggs used by silkworm farmers in South Sulawesi Province, which come from the production of Perum Perhutani (KPSA Soppeng) and are also imported egg seeds from China [19,95].

The price of eggs from Perhutani is more affordable for silk farmers with relatively good productivity, although the production quality is unstable due to changes in weather conditions. The production of silkworm eggs from China is higher than that of *Perhutani* eggs [94,96], but it is only available in certain months [19]. Chinese eggs have higher adaptability and steadiness in yield than hybrid C301, but hybrid C301 showed better cocoon and yarn quality in the observed parameters. Importing silkworm eggs must be balanced with the need for adequate storage equipment, considering that the import of eggs is carried out in large quantities and is not directly absorbed by the market. Poor egg storage results in many eggs dying, hatching not being uniform, and the larvae of the following generation being weak [77,97].

Another problem with silkworm eggs is related to the limitations of the center and breeders. Up until now, there has been only one breeder operating (KSPA Soppeng) with a limited capacity. The limited number of breeders impacts the use of silkworm eggs in rearing locations that are far from the nursery location. With the lack of activity of the Candiroto Silkworm Breeding Center (PPUS), silkworm farmers in West Java and surrounding areas obtain silkworm eggs from Soppeng’s KPSA in South Sulawesi, thus requiring a lengthy shipping process. The time the eggs are in transit increases the risk of egg damage.

The PS 01 hybrid production is a solution for the existence of superior silkworm eggs. Unfortunately, PS 01 silkworm eggs are not yet on the market [98]. The Directorate of Social Forestry and Customary Forest Business Development (Bina Usaha Perhutanan Sosial & Hutan Adat/BUPSHA), part of the General Directorate of Social Forestry and Environmental Partnership (PSKL) in collaboration with the Center for Forest Research and Development of Forest, has created Bina Mandiri Farmer Group (BMFG) to become the PS 01 breeder, functioning as a supplier of silkworm eggs for the Sukabumi Regency and surrounding areas [98]. However, until now, it has not been produced optimally.

#### 2.2.3. Silkworm Rearing

Silkworm rearing is a series of activities to produce cocoons using silkworm eggs with certain cultivation standards. The success of cocoon production is highly dependent on compliance with its SOPs, temperature, and humidity, and the condition of the administered leaves [99]. Sericulture farmers in Sukabumi have good knowledge of silkworm rearing standards [100], but many do not apply them, resulting in low or even negative income [79]. Support from Sericulture Laboratory technicians of the Forest Research and Development Center for applying good silk-rearing standards in Forest Management Unit/FMU (Kesatuan Pemangkuan Hutan/KPH) Boalemo has resulted in higher cocoon productivity than that of FMU Yogyakarta without assistance [101]. Therefore, efforts are needed to increase farmers’ adoption of good rearing standards by providing consistent, accessible, relevant, and practical information with more personal means of communication and stakeholder collaboration, using an interdisciplinary approach including social scientists, communication specialists, and marketing experts [102].

People who still cultivate silkworms at home can potentially interfere with silkworm cultivation activities [41]. *B. mori* L. is very delicate, highly sensitive to environmental fluctuations, and unable to survive extreme conditions [103]. Thus, traditional rearing activities carried out at home will result in low cocoon productivity.

**Figure 4:**
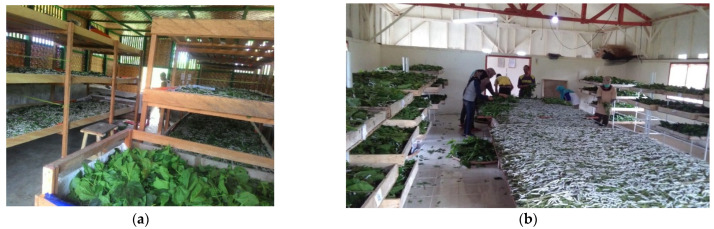
Silkworm rearing in Indonesia. (**a**) South Sulawesi Province; (**b**) West Java Province.

Complete and appropriate infrastructure facilities (separate rearing houses for first and last instar silkworms, mulberry fields) are important for silkworm rearing. They will affect the number of rearing cycles in a year. Silk cultivation in FMU Boalemo can be done 12 times per year with appropriate facilities and infrastructures in one location. This differs from the BMFG in Sukabumi, which can only cultivate eight cycles in one year and keeps young silkworms and grown silkworms separate. More rearing cycles will increase yield productivity. Silkworm rearing in Indonesia can be seen in Figure 4.

#### 2.2.4. Pests and Diseases

Sericulture in Indonesia has experienced two waves of epidemics of pebrine disease and Nuclear Polyhedrosis Virus (NPV) infection, causing a drastic decline in sericulture production [21]. Both mulberries and silkworms are infested by a number of pests, which affect the cocoon quality and productivity, resulting in economic losses for farmers [104]. Among the silkworm diseases that cause economic damage, the highest crop loss is attributed to viral diseases, accounting for 70% of the total loss of the crop every year [105].

Diseases that often attack silkworms in Indonesia are NPV, Infectious Flacherie (FV), Aspergillus, Muscardine, pebrine, bacteria, and poisoning from agricultural chemicals [21,106,107]. The causes of these diseases include bacteria, fungi, viruses, and physical and chemical factors as well as environmental factors that result in abnormal conditions and death [108].

### 2.3. Silkworm Products

#### 2.3.1. Main Products

**Figure 5:**
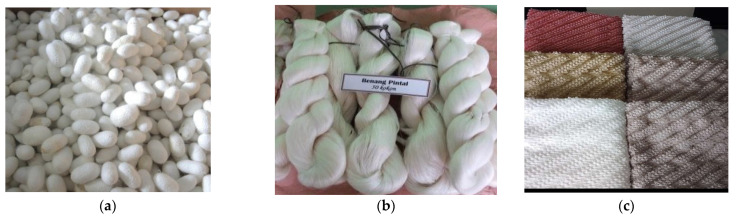
The main products of sericulture. (**a**) cocoons; (**b**) yarns; (**c**) material fabrics.

The main products of sericulture are cocoons, yarns, and material fabrics (Figure 5). The trend of cocoon production [72] and yarn production [109] continues to decline. In the 1990s, Indonesia’s silk yarn production reached 120–140 tons/year. Nowadays, it is only around 2.5 tons/year [20]. The current national demand for silk yarn is 900 tons per year [110], and it is predicted to increase by 12.2% annually [72]. The solution to the scarcity of silk yarn supply is generally importing. As stated by Antaranews [110], 95% of Indonesia’s silk yarn is still imported from China, India, and Japan. Therefore, Indonesia has changed its sericulture strategy for the export market to meet domestic needs [111].

- Cocoons

Cocoons are the final product of silkworm rearing. The quality of the cocoons is determined by the hereditary nature of the silkworm strains and environmental conditions during rearing, spinning stage, etc. [112]. The requirements of a perfect cocoon are healthy (no defects), clean (clean white), the inside (pupa) not damaged or crushed, and a hard silk shell (layer of silk fibers) resistant to finger pressure. Several parameters of cocoon quality affect the quality of the raw materials for silk fiber and determine the quality, quantity, and efficiency of the process [113]. Cocoon characteristics, such as cocoon weight, cocoon shell weight, and cocoon shell ratio, are the most important economic determinants of silkworm rearing [114]. Different shapes and sizes of cocoons will produce various sizes of silk fiber and yarn quality.

The quality of the C301 hybrid showed higher yields compared with hybrids from China with respect to these parameters: percentage of normal cocoons, the weight of cocoons, weight of cocoon shells, and proportion of cocoon shells [77]. China’s normal hybrid cocoons are 80.08–90.08%, while the C301 hybrid is 86.75–93.25%. The weight of the Chinese hybrid cocoon is 1.44–1.57 g, while that of the C301 hybrid is 1.7–2.04 g. The weight of the Chinese hybrid cocoon shell is 0.3–0.32 g, while the C301 hybrid is 0.41–0.46 g. The percentage of Chinese hybrid cocoon skin is 20.58–20.96%, while that of the C301 hybrid is 22.94–23.97% [77].

Hybrid PS 01 (Chinese race male and Japanese race female—(804 × 927)) has an egg hatching percentage of 97.00% compared with hybrid C301, which is only 96.01%. Hybrid PS 01 produces a cocoon weight of 1.6 g, cocoon shell weight of 0.29 g, and a cocoon shell ratio of 20.35–21.36%, the hybrid C301 cocoon weighs 1.27 g, the cocoons shell weighs 0.2 g, and the cocoon shell ratio is 17.9–19.54% [39].

The cocoon shell percentage is one of the benchmark measures for determining the selling price of cocoons. The highest level (grade of cocoon quality) is a percentage of cocoon shells of 22–25% [115].

High competition with producers in other countries, low government support for this industry, lagging technology, and poor handling of pests and diseases are thought to hinder the development of silkworm cultivation in Indonesia. In 2020, globally, Indonesia’s silk production was only twelfth behind China, India, Brazil, Thailand, and Japan [20]. From year to year, Indonesia has experienced a decline in cocoon production.

- Silk Yarn

The quality of silk fiber is very important in the reeling process because it will affect the silk yarn obtained. These qualities include the dry cocoon weight, number of fiber breaks, remaining cocoon, percentage of fiber weight, and reelability (fiber unwinding). Reelability is the ability of silk fibers to unravel and coil when the cocoon is spun. Cocoon is highly dependent on the silkworm variety, temperature, and humidity during spinning. The cocoon mountages also affect the cocoon unraveling capacity [60].

Silkworm eggs from KSPA Soppeng and PPUS Candiroto, when maintained in conditions of high temperature and low humidity, did not show any difference in a produced cocoon. Still, there was a difference in the filament yield. Longer filaments were produced by silkworm eggs from KSPA Soppeng compared with the eggs from PPUS Candiroto [35].

The quality of the C301 hybrid filament is better than that of the Chinese hybrid for the parameters of filament length, percentage of filament, denier thickness, and reelability. The China hybrid filament length is 889.08–994.84 m; the filament percentage is 17.91–19.14%, the denier thickness is 2.37–2.97, the reelability is 85.57 –95.56, and the yarn weight per 50 cocoons is 8.87–10.7. The C301 hybrid produces a filament length of 1026.05–1127.83 m, filament percentage of 21.24–21.49%, denier thickness of 2.71–3.63, reelability of 76.11–89.58, and yarn weight per 50 cocoons of 9.51–13.66 [77]. Hybrid PS 01 (Chinese race male and Japanese race female—(804 × 927)) produced a filament length of 1003.67–1160 m, 32% longer than the C301 hybrid, which only had a filament of 755.67–883.33 m [39].

To encourage the development of the national silkworm industry, the Government of Indonesia (GOI), through the Ministry of Forestry, Ministry of Industry, and Ministry of Cooperatives and Small and Medium Enterprises—issued Joint Regulation No. P47/Menhut-II/2006, No. 29/M-IND/PER/6/2006 and No. 07/PER/M.KUKM/VI/2006 concerning the Guidance and Development of National Natural Silk with a Cluster Approach [27]. Through this joint regulation, the government plans to build an integrated silk industry cluster from upstream to downstream, including cultivating mulberries, silkworms rearing, and cocoons for the yarn and silk reeling industry. Furthermore, to encourage the above efforts, the Ministry of Forestry issued Ministerial Regulation no. P.56/Menhut-II/2007 concerning the Procurement and Circulation of Silkworm Eggs.

Ironically, when the cluster effort was launched (2006), the annual national silk yarn production fell to less than 32 tons and the yearly demand for domestic industry to around 700 tons [116]. On the other hand, at the global level, the market for silk yarn in 2005 continued to increase, reaching the demand of 100,000 tons. Meanwhile, to fill the shortage of domestic silk yarn, the government allowed imports of silk yarn of more than 600 tons from China, India, Thailand, and other ASEAN countries [116,117]. The imports from silk yarn-producing countries turned out to harm the national silk yarn industry. Domestic artisans prefer imported yarn, especially from China, because the price is lower. For silk entrepreneurs, the presence of spikes yarn or synthetic yarn at a low price affects the selling power of natural silk fabrics. Thus, lower-class people especially prefer it because the price is low [118].

- Material Fabrics

**Figure 6:**
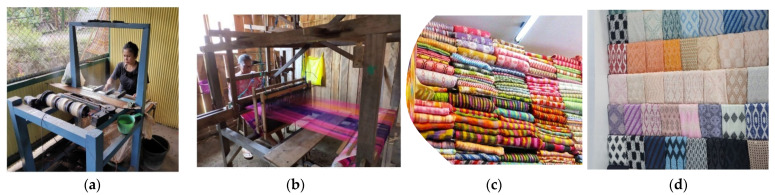
Process and material fabrics. (**a**) spinning; (**b**) weaving; (**c**) Sulawesi woven fabrics; (**d**) West Java woven fabric.

Spinning and weaving will produce material fabric. Spinning is the process of extruding cocoon fibers into spun strands. There are two categories of spinning, modern spinning and people spinning. Modern spinning is spinning that uses machines, both fully automatic and semi-automatic. People’s spinning machines in South Sulawesi have three stages in the spinning process, and all stages affect the quality of the yarn. The three stages are the process of cocoon handling, spinning, and yarn handling spinning result [40]. The patterns of woven fabrics are produced based on regional characters. The results of woven fabrics from two areas of silk development in Indonesia can be seen in Figure 6.

#### 2.3.2. Diversification Product

Despite the aforementioned challenges, the untapped potential of silk-based products makes us optimistic. Beyond conventional textiles, there are many opportunities for product diversification. This is due to the fact that silkworm cocoons and pupae can be exploited as sericin [119], regenerated silk fibroin [120], and chitin/chitosan [121]. These are high-value biomaterials with potential applications in many fields. In the last couple of decades, progress around the science of silk has enabled the utilization of silk-based materials in exciting areas such as the pharmaceutical/medical field [121,122,123,124], cosmetics [125,126], electronics [127,128,129], the energy sector [130,131]. Although these are niche markets, product diversification efforts may lead to renewed interest from investors and developers in the sericulture industry. To advance in this direction, it is important for the scientific community to collaborate with farmers in the hope that high-value niche products may give some additional competitive advantage against other commodities at both national and global scales.

### 2.4. Social, Economic, Cultural, and Institutional Factors

#### 2.4.1. Social, Economic, and Cultural Factors

Sericulture plays an important role, especially in rural communities [109,132]. The sericulture industry involves minimal investments, a short cultivation period, high employment potential, and very profitable returns that are suitable for the economy of a rural agrarian community [133,134].

Natural silk is a labor-intensive industry and provides jobs for many people [135]. The proportion of labor costs for silk cultivation reaches 73.14% [85]. Sericulture business actors, especially the upstream sector in Indonesia, are still dominated by people with low levels of education, mostly at an elementary school level [17,44,79,135]. However, some of them have a high school or undergraduate education [41,136], and the majority are aged between 15 and 64 years [137]. Even though education is not the main requirement [44], the sustainability of silk needs to be supported by an adequate level of education to update technical knowledge, management, and mindset [138] in the adoption of technology for a commercial business. In its implementation, the upstream sector involves a lot of family labor and does not use wage labor, so it is more of commercial household business.

Of 9223 Business Farmer Groups in the Social Forestry unit, only 27 focus on sericulture development (West Java, North of Kalimantan, and South Sulawesi); 23 focus on mulberry and cocoon production, and 4 on silk processing [139]. Farmers carry out sericulture cultivation as a primary or secondary job [44]. The average income of cocoon farmers is Rp. 2,383,969 (USD 156.56) per head of family/year, while the average income of yarn farmers is Rp. 8,004,500 (USD 525.68) per family head per year [140]. The income showed the shrink of the business and led these activities to be part of side occupations which contribute to the additional income for the family.

There is a decreasing number of silk farmers as they switch to other commodities [40]. An insufficient number of eggs was one of the main problems. On the other hand, if enough eggs are produced, the quality is sometimes not so good. Hence, the production will fail and cause uncertainty in the cocoon yields. The minimal number of cocoons produced affected the availability of the raw materials for weavers [141].

Women play an important role in natural silk cultivation [142,143]. According to [17,143,144]. silk activities involve distributed gender roles through internal agreements [145,146], and this condition is different in each region [40]. Men are more focused on mulberry cultivation and leaf harvesting, which require physical energy, while women are dominant in rearing, feeding, harvesting cocoons, weaving, and reeling, which require patience [142,147]. Some rural women consider silk activities as their primary or secondary livelihood. The distribution of internally agreed roles involves substantial social capital in supporting the future development of labor-intensive sericulture [147].

Processing cocoons into yarn and silk is characterized as a traditional household business [148]. The craft of silk weaving was initially to meet the family’s needs and turned into a commercial enterprise with a cluster of handicraft industries. However, traditional characteristics remain attached to simple reeling (non-machine looms). In South Sulawesi, there are around 4900 silk woven fabric artisans that produce 99,000 sarongs per year; 227 of them are non-machine loom craftsmen with a fabric production of 1,580,000 m per year [22]. Thus, the silk business is a cultural asset of the Indonesian nation that must be maintained and preserved.

The foremost opportunity for the development of Indonesian silk is the culture of the people in the use and management of silk production [136]. Silk cloth became a traditional costume and a symbol of social status. Silk is used at traditional, religious, and official national events [40,44] and is a mainstay commodity and souvenir typical of South Sulawesi [41]. Silk motifs and creations also represent the wearer’s position, happiness, and safety [149].

#### 2.4.2. Sericulture Development Policy

The policy of developing silk in Indonesia aims to encourage agribusiness to produce silk products of good quality in large quantities to meet the demands and standards of the domestic and export markets [24,25]. Sericulture was first developed in South Sulawesi Province through the cooperation of the Directorate General of Forestry, Ministry of Agriculture, with the Japan International Cooperation Agency (JICA).

In 2002 through Forestry Minister Decree No. 664/Kpts-II/2002, a Natural Silk Center was formed to support the development of silk in Indonesia. Balai Persuteraan Alam (BPA) has a vast working area, covering almost the entire territory of Indonesia with natural silk development activities, starting from institutions arrangement and skills development through training to data collection from upstream to downstream. Silk development refers to the Joint Regulation of the Minister of Forestry Number P.47/MENHUT-II/2006; the Ministry of Industry Number 29/M-IND/PER/6/2006, and the State Ministry of Cooperatives and Small-Medium Enterprises Number 07/PER/M.UMKM/VI/2006 concerning the Guidance and Development of National Natural Silk through a cluster approach. Business development with a cluster system (upstream-downstream) was developed through a partnership scheme. In this scheme, business groups engaged in the sericulture business (mulberry farmers, silkworm cultivators, and silk yarn industries) and related institutions are geographically close together.

At the provincial level, the government of South Sulawesi launched the “Restore the Glory of Silk” program to recreate the level of Indonesian silk production achieved in the 1970s. In order to support this agenda, the South Sulawesi Regional Government has developed various programs to strengthen the upstream and downstream sectors, including technical assistance, equipment, marketing, farmer empowerment, and capital [41]. To improve the quality and quantity of silkworm eggs and ensure the quality and availability of silk cocoons, in 2017, the Ministry of Forestry launched MoF number P.37/MenLHK/Setjen/Kum.1/6/2017, which regulates the procurement and circulation of silkworm eggs.

In 2016, there was an institutional change at BPA to become the Center for Social Forestry and Environmental Partnerships (Balai Perhutanan Sosial dan Kemitraan Lingkungan/BPSKL) through the Regulation of the Minister of Environment and Forestry of the Republic of Indonesia Number P. 14 /Menlhk/Setjen/OTL.O/I/2016.

The development of the silk agro-industry in Indonesia is influenced by changes in policies related to institutions. Starting in 2016, the silk agro-industry development activities became part of the social forestry (PS) activities supported by the Decree of the Minister of Environment and Forestry No. 83/2016 concerning Social Forestry, which provides access rights for communities in forest management areas. However, silk activities have become limited because they are only a small part of social forestry. They are, therefore, only supported by 1.2–1.5% of the total budget of BPSKL.

Fortunately, in 2020 the GOI stipulated Law Number 11 of 2020 concerning Job Creation and Government Regulation (PP) and its derivatives Number 23 of 2021 concerning forestry administration which opened up opportunities for forest areas to permit holders to develop multiple forestry businesses, including the development of silk cultivation through Forest Utilization Business Permits (PBPH). The dynamics of government policies that affect the development of the silk agro-industry in Indonesia can be seen in Supplementary Table S2.

#### 2.4.3. Institutional Factors

Sericulture agribusiness is carried out by three leading players, namely the public sector (government institutions, both central and regional), the private sector (legal entity organizations), and farmer groups [150]. The public sector has a role in setting regulations [150] to ensure mulberry availability and quality, the provision of silkworm technical guidance, evaluating facilities and infrastructure, increasing human resources, and updating databases [40]. Private sector groups play a role in terms of providing capital, distributing various inputs needed in mulberry and silkworm cultivation (fertilizer, mulberry, and silkworm planting material), the yarn spinning industry, and marketing the products. The farmer group has a role as a mulberry and silkworm cultivation actor in producing cocoons.

These three stakeholders are involved in the upstream and downstream activities of sericulture. The parties involved are as follows: (a) Upstream Sector: public groups (BPA/BPSKL, Provincial Forestry Service, District Forestry Service), private groups (Perum Perhutani and BMFG, which produces silkworm), mulberry farmer groups and silkworm farmers, (b) Downstream Sector: public groups (Provincial Industry and Trade Office, Regency Industry and Trade Office), Business Groups (PT. Kokon Sutera Sulawesi*);* and (c) Supporters (financial institutions, Forest Research and Development Agency, Industry & Trade Research Institute, colleges).

The parties involved in silk activities have different organizational structures and rules [151]. From 2006 until 2012, natural silk still relied on cluster policies [27]. During this period, institutional development was carried out by the Ministry of Environment and Forestry, the Ministry of Industry, and the Ministry of Cooperatives and Small Enterprises. However, in reality, these three institutions are still running independently [27]. This shows that there is a fragmentation between upstream and downstream in the management of Indonesia’s silk, which has the potential to weaken efforts toward improvement.

The motivation for establishing silk institutions at the upstream level was different in the silk centers of Indonesia. The establishment of silk institutions in West Java and Central Java was generally initiated by the national reforestation program. On the other hand, the silk farmers ‘institutions in South Sulawesi were formed on the motivation of the socio-cultural attachment of the community to the use of silk fabrics. This has induced Wajo farmers to be more independent. This condition is different from silk farmers in West Java, who still depend heavily on assistance from other parties [100].

The institutional capacity of the sericulture business in Indonesia is generally at a small scale, even at the household level, and only reaches the silkworm cocoon stage. Small-scale development, only up to the cocoon production stage, causes the bargaining position of farmers to be relatively weak, with prices determined by buyers [85]. Widiarti et al., [152] found that the basic problems of small businesses such as sericulture are a lack of management skills and professionalism, as well as limited access to capital, technological innovation, and marketing networks. These challenges have hampered institutional performance, especially at the farmer level, and often led to the reluctance of large companies to cooperate.

The change in the centralization of the forestry sector from regencies to provinces has also disrupted the partners of natural silk farmer groups in the field [40]. The ongoing changes in government institutions hamper the sustainability of the silk program, such as the lack of control in the development of techniques, infrastructure, human resource development, and a database that supports decision-making. In addition, public sector institutions that handle silk often change their functions. The Natural Silk Center, as one of the technical implementing units of the Ministry of Environment and Forestry that is specialized in handling natural silk, has turned into a Social Forestry and Environmental Partnership Center (BPSKL), whose main task is not only dealing with silk. This change aims to simplify the structure but is affected functionality. However, the problem of silk is very complex (upstream and downstream, from mulberry to yarn), so organizational changes are not sufficient. One of the consequences of these institutional changes is the loss of information (in the term of database) on silk production, such as production potential, demand, the number of parties involved, the area used for mulberry cultivation, etc.

## 3. The Way Forward

### 3.1. Moriculture

Based on the existing conditions of natural silk related to the fulfillment of silkworm (*B. mori* L.) feed sources, the main problem was the low and discontinuous productivity of mulberries. The food availability factor is important since silkworms are monophagous; hence their entire life cycle depends on mulberry leaves [153]. However, the tropical climate in Indonesia causes the productivity and supply of mulberry leaves to be discontinuous [28,66].

Efforts that can be made to overcome the problems with mulberry productivity as a source of silkworm feed include:

1. Enhance the commitment and compliance of all related stakeholders to apply standard operating procedures in cultivating mulberries (selection of suitable locations for mulberry cultivation, cultivation techniques, intensive rearing, appropriate fertilization, and preventive measures to anticipate plant pests and diseases).
2. Superior mulberry seeds with high productivity that can be planted under various growing conditions. Indonesia, through the Ministry of Environment and Forestry, has initiated and produced a mulberry hybrid with productivity of 52.35 tons/ha/year [39].
3. Integrating mulberry agroforestry with other plants to increase land productivity and mulberry production in Indonesia. Agroforestry that integrates mulberry plantations with agricultural crops provides multidimensional benefits, including the efficient use of land area, increased production and total leaf yield, improved nutrition, efficient use of light and water from the mulberry plant, and its inherent ability to combat pests and diseases [46]. Expanding the mulberry plantation to the potential of the Social Forestry areas of 5.03 million Ha [139].
4. Managing mulberry blocks to ensure the continuity of mulberry production. A good field division will optimize feed production so that, in addition to optimal leaf quality and productivity, it can also increase the intensity of silkworm rearing per year. The separation of young and grown silkworms into different facilities with the different dedicated mulberry fields has the advantages of intensive maintenance, pest control, and feed productivity prediction, considering that young silkworms need one-month-old mulberry feed. In contrast, grown silkworms require two months old mulberry feed. Otherwise, it will cause losses due to separation, which will result in high maintenance operational costs and will require a wider allocation of the mulberry plantation area.

### 3.2. Silkworm Cultivation (Sericulture)

One of the main obstacles to sericulture development in Indonesia is the absence of quality silkworm eggs and the application of silkworm rearing that does not follow the appropriate procedures. Silkworm egg producers are still limited with low production. Some of the efforts that can be made include:

1. Creation of silkworm breeding centers as silkworm egg producers with superior strains of silkworm eggs. The scheme for establishing a silkworm nursery center in collaboration with related stakeholders in Sukabumi can be an example to be implemented in other areas, especially in South Sulawesi.
2. Develop superior silkworm hybrids. The Ministry of Environment and Forestry has initiated the distribution of the superior silkworm hybrid strains PS 01, which has 35–40 kg/box cocoon production. This strain can be developed more widely to increase the productivity of Indonesian sericulture.
3. Enforcement in implementing SOP for silkworm rearing. The SOP for maintaining silkworms that are not carried out properly has an impact on the productivity of cocoons and yarns. Adherence to selecting a good and suitable location (effectiveness of feed sources and minimal air pollution), carrying out room and silkworm disinfection procedures, and paying attention to the timing and rearing procedures correctly will be steps to ensure optimal cocoons and yarns.
4. The separation of young and grown silkworm farmers can increase productivity. Young silkworms require intensive care and are susceptible to disease. Productivity will increase if skilled farmers carry out the maintenance of young silkworms. Only then can the maintenance of grown silkworms be carried out by novice farmers. The advantages of this separation are the effective use of the rearing room, and the productivity is maintained because young silkworms are susceptible to disease, so they will only be kept by skilled farmers. Good cooperative arrangements can increase the intensity of maintenance. Disadvantages will arise if communication is established between young and grown silkworm farmers; high mobility is also required because of the distance between the two.

### 3.3. Products

The main products of sericulture are cocoons and yarns. So far, the quality of the cocoons produced does not match the market or consumer standard, which has an impact on yarn imports and causes competition with synthetic yarns such as rayon and cupro. A strategic step to overcome this problem is the selection of cocoons at the time of silkworm rearing so that the quality is optimal and the price can be higher. Supposing the requirement for quality silk yarn can be appropriately fulfilled. The problem of competition with synthetic yarns can be avoided, considering that the existence of synthetic yarns is due to the unavailability of raw materials for silk yarns. Silk’s loyal consumers have a different market share; hence, they will not be affected by synthetic yarns. Moreover, the cultural factor in South Sulawesi, which requires the use of silk, means the silk market can be maintained.

### 3.4. Social, Economic, and Cultural Factors

Sericulture can increase people’s income, absorb labor, and be applied in rural areas. Livelihoods in the sericulture sector are discontinuous due to the absence of eggs causing many people to turn to other commodities. Some efforts can be made to tackle the situation:

1. Build production networks and cooperate with stakeholders, facilitated by the government to ensure the sustainability of the production of superior eggs and the propagation of silkworm egg producers.
2. Internalization of silkworm rearing (sericulture) through both formal and informal education involving various stakeholders, including business stakeholders, to maintain socio-cultural values and develop the value of silk entrepreneurship as part of the nation’s identity.
3. Increasing the community’s capacity for silkworm rearing in terms of technical aspects, farm management, farmer group organization management, and improved access to markets. The yield of silk products is low due to a lack of assistance regarding proper rearing procedures. Efforts are needed to increase farmers’ adoption of good rearing standards by providing consistent, accessible, relevant, and practical information via more personal means of communication and coupling with stakeholders by a transdisciplinary approach, including social scientists, communication specialists, entrepreneurs, and marketing experts. The role of the Forest Management Unit/FMU, which is the implementation of management at the site level, needs to be more intensive for facilitating sericulture development.

### 3.5. Institutional Factors

One important factor that needs to be considered in the institutional system is the arrangement between rights and obligations contained in a mutually binding agreement. One example is the collaboration between silk farmer groups in Sukabumi with PT. Begawan Sutera Nusantara (PT. BSN) in terms of joint utilization of production activities that can increase competitive advantage through capacity utilization and product diversification [85,152]. Shared use arises from the opportunity to share activities (PT BSN, which carries out the egg production process and raises young silkworms, is a producer of eggs for young silkworms, while farmers are rearing grown silkworms and harvesting cocoons that are taken and paid for by PT BSN), similarities in technology (assistance is provided by the company), distribution or other factors. The joint use of production activities can increase utilization capacity and product diversification and, in turn, achieve economies of scale. This will increase the productivity and competitiveness of sericulture. Improving the ability to build networks, increasing social capital within the group, and improving the organizational management and negotiation skills of farmers are important factors in supporting silk farming institutions. In addition, local governments can provide formal support through local regulations, policies, and silk development programs at the local level.

## 4. Conclusions

Sericulture in Indonesia involves the production of non-timber forest products and has been practiced for generations by the local community for their livelihood. However, there has been a decrease in this activity over the last 5–10 years. There are various and complex problems from upstream to downstream that have led to this unfortunate development. Moriculture and sericulture techniques, the quality and quantity of the products, the social-economic aspect, institutional arrangements, and community motivation are interconnected. They have resulted in a challenging atmosphere for sericulture development. On the other hand, there are potential resources such as exploring qualified mulberry production and quality silkworm production through research and development, valuable cultural aspects, and potential stakeholders for building network engagement. The commitment, cooperation, and action of all stakeholders are needed to improve sericulture development in Indonesia. In this context, the central government can play an important role in facilitating multi-stakeholder partnerships in integrated sericulture development in Indonesia.

## Figures and Tables

**Table 1:** The productivity of mulberry hybridization in Indonesia.

The Origin of the Cross	Leaf Production (ton/ha/year)
*M. australis* Poir *x M. indica* L.	23.2 *
*M. nigra* L. *x M. indica* L.	23 *
*M. cathayana* Hemsl. x *M. amakusaguwa (Hybrid M. bombycis* Koidz *x M.s acidosa* Griff*.)*	52.35 **
*M. acidosa* Griff*.x M. latifolia* Poir.	35.42 **
*M. australis* Poir *x M. indica* L.	29.56 **

Source: * [62], ** [63].

**Table 2:** The productivity of silkworm eggs in Indonesia.

Egg Type	Producer	Hatching Percentage (%)	Characteristics	Cocoon Quality	Filament Quality
C301 *	KSPA Soppeng and PPUS Candiroto	81.65–81.95	- egg color: gray - eggshell color: white - silkworm pattern: spots - silkworm segment color: gray - silkworm life: 24–26 days - silkworms cannot stand being kept in standard conditions - The cocoon is white and oval shape	- Normal cocoons: 86.75–93.25% - Weight of cocoon: 1.7–2.04 g - Weight of cocoon shell: 0.41–0.46 g - Cocoon shell ratio: 22.3– 23.97%	- Filament length: 1026–1127 m, - Filament percentage: 21% - Filament denier: 2.71–3.63 - Decomposition: 76–89%
BS 09 **	KSPA Soppeng dan PPUS Candiroto	>90	- egg color: gray - eggshell color: white - silkworm pattern: spots - color between segments: blue - silkworms are somewhat sensitive to minimum conditions - cocoon shape: oval - cocoon color: white	- Normal cocoons: 90.00–96.00% - Cocoon shell ratio: 21.28–23.49%	- Filament length: 1060–1216 m, - Filament percentage: 16.64–19.09% - Filament denier: 2.3–3.26
PS 01 ***	Bina Mandiri Forest Farmers Group, Sukabumi	90.74–96.99	- egg color: gray - eggshell color: white - silkworm pattern: spots - silkworm color between segments: slightly bluish gray - silkworm growth is more uniform - resistant to disease - the life of the silkworm is 25–27 days - cocoon color: white - stable cocoon shape	- Normal cocoons: 97.5–100% - Weight of cocoon: 1.7–1.9 g - Weight of cocoon shell: 0.38–0.44 g - Cocoon shell ratio: 22.29–24.82%	- Filament length: 808–1003 m, - Rolling power: 95–100% - Filament denier: 2.3–2.43 - Spun yield: 13.29–15.31

Source: * [77], ** [91], *** [87].

## Data Availability

Not applicable.

## Supplementary Materials

The following supporting information can be downloaded at: https://www.mdpi.com/article/10.3390/insects13100913/s1, Table S1: Silkworm hybridization in Indonesia, Table S2: The dynamics of government policy affecting silk development in Indonesia.
