# Supplementary material for: Lens on Tropical Sericulture Development in Indonesia: Recent Status and Future Directions for Industry and Social Forestry
Source: Insects. 2022 Oct 8;13(10):913. doi: 10.3390/insects13100913 (PMC9604369; doi:10.3390/insects13100913)
Supplement: Supplementary file 1 [file insects-13-00913-s001.zip › insects-1913626-supplementary.pdf]

Table S1. Silkworm hybridization in Indonesia.

Product Name	Breeding	Breeders	Advantages	Recognition as intellectual property rights
Hybrid C-301	Chinese race male and Japanese race female – (202 x 102)	PPUS Candiroto (Pati, Central Java)	Adapts well to varoius climatic conditions; filament size: 758.7–904.2 m with a cocoon weight 1.39–1.48 g [52,75,77,86]	The superior seeds are traded by Perum Perhutani, but no intellectual property rights are issued.
Hybrid BS-08	Japanese race male and Chinese race female – (805 x 806)	Sericulture Laboratory, Research and Development Agency, Ministry of Forestry	High cocoon shell ratio with hatchability above 90%, filament size 1002−1251 m and cocoon shell ratio 23.00−25.04%	Decree of the Minister of Forestry No. SK. 369/Menhut-VIII/2004 on October 8, 2004
Hybrid BS-09	Chinese race male and Japanese race female – (808 x 807)	Sericulture Laboratory, Research and Development Agency, Ministry of Forestry	Has good cocoon quality and filament strength and hatchability above 90% with a filament size of 1060 −1216 m and a cocoon shell ratio of 21.28 – 23.49%. This hybrid is more resistant to pebrine disease and easy to maintain	Decree of the Minister of Forestry No. SK. 369/Menhut-VIII/2004 on October 8, 2004
Hybrid PS 01	Chinese race male and Japanese race female – (804 x 927)	Sericulture Laboratory, Research and Development Agency, Ministry of Forestry	Good and stable cocoon quality (cocoon shell weight: 0.38–0.44 g); high egg hatchability, more than 90%; good-quality filaments (filament size 808–1003 m) and also suitable for cultivation in high places [39]	Minister of Forestry Decree no. SK 794/Menhut-II/2013 dated November 13, 2013
Hybrid SINAR	Chinese race male and Japanese race female – (804 x 102)	Sericulture Laboratory, - Forest Research, Development and Innovation Agency, Ministry of Environment and Forestry	Filament size up to 1102 m, suitable for maintenance in lowland areas with an altitude of 100−200 m above sea level	Decree of the Minister of Environment and Forestry of the Republic of Indonesia No: SK.300/Menlhk/Setjen/KUM.1/4/2019 dated 24 April 2019

Table S2. The dynamics of government policy affecting silk development in Indonesia.

Year	Policy	Description
1986	Minister of Forestry Instruction No. 02/Menhut-II/86 dated January 3, 1986	The Crash of the Natural Silk Handling Program in South Sulawesi Province
2002	Decree of the Minister of Forestry No. 664/Kpts-II/2002 dated March 2, 2002	The Organization and Work Procedure of the Natural Silk Center.
2006	Joint Decree of 3 Ministers No. P.47/Menhut-II/2006 29/M-ENG/PER/6/2006 07/PER/M.KUKM/VI/2006	Fostering and Development of National Natural Libraries with Cluster Approach
2016	Regulation of the Ministry of Environment and Forestry of the Republic of Indonesia Number P. 14 /Menlhk/Setjen/OTL.O/I/2016	Organization and Work Procedures of Balai Social Forestry and Environmental Partnership
2017	Regulation of the Ministry of Environment and Forestry of the Republic of Indonesia Number P.37/MenLHK/Setjen/Kum.1/6/2017	Improving the quality and quantity of silkworm eggs, ensuring the quality and availability of silk cocoons, regulating the procurement and circulation of silkworm eggs
2021	Government Regulation (PP) and its derivatives Number 23 of 2021	Concerning forestry administration, it opens opportunities for forest area permit holders to develop natural silk with multiple forestry businesses through Forest Utilization Business Permits (PBPH) in one forest landscape
2021	Regulation of the ministry of the environmental of Social Forestry of Indonesia Number 09 of 2021	Concerning Social Forestry, it enables a Farmer Business Group of Mulberry growers and the development its derivative products
